# Factors influencing turnover intention among primary care doctors: a cross-sectional study in Chongqing, China

**DOI:** 10.1186/s12960-018-0274-z

**Published:** 2018-02-13

**Authors:** Tong Wen, Yan Zhang, Xue Wang, Guo Tang

**Affiliations:** 1 School of Public Health and Management, Chongqing Medical University, Chongqing, 400016 China; 2 Research Center for Medicine and Social Development, Chongqing Medical University, Chongqing, 400016 China; 3 Innovation Center for Social Risk Governance in Health, Chongqing Medical University, Chongqing, 400016 China

**Keywords:** Turnover intention, Job satisfaction, Doctor, Primary health care, China

## Abstract

**Background:**

The intention to leave a job, known as turnover intention, among primary care doctors has a significant impact on primary health care service delivery. We investigated primary care doctors’ turnover intention and analysed associated factors involved in primary health facilities in Chongqing, China.

**Methods:**

A total of 440 doctors were interviewed, they were selected using a multi-stage stratified random sampling method. The survey instrument was a self-administered questionnaire which assessed socio-demographic and work-related characteristics, job satisfaction and turnover intention. The data were analysed using *χ*^2^ test, one-way analysis of variance, exploratory factor analysis and linear regression analysis.

**Results:**

Our study found that 42.3% of the primary care doctors we sampled in Chongqing, China, intended to resign. Location, age, job title, doctor’s position level, work pressure and job satisfaction were associated with turnover intention. Job satisfaction included both *employment-related job satisfaction* (including “your chance of promotion”, “your rate of pay” and two other items) and *satisfaction with the job itself* (including “the freedom to choose your own method of working”, “your job safety” and two other items).

**Conclusions:**

Improving job satisfaction, in terms of salary, promotion and job safety, is crucial for reducing turnover intention among primary care doctors. Therefore, we suggest that the government increase its financial investment in primary care facilities, especially in less-developed areas, and reform incentive mechanisms to improve the job satisfaction of primary care doctors. The government should consider policies such as establishing a social pension programme for village-level doctors and providing more opportunities for job promotion among primary care doctors, especially township-level doctors. Attention should also be paid to the impact of rapid urbanization, which could lead to increased workload or increased opportunities for career development, thus affecting primary care doctors’ turnover intention.

## Background

Primary health care (PHC) is pivotal now more than ever [1]. The demand for PHC is expected to increase considerably due to an ageing population with more chronic diseases [2]. However, primary care facilities are facing significant labour shortages worldwide [3]. According to the Association of American Medical Colleges (AAMC), the USA will face a shortage of between 7300 and 43 100 primary care physicians by 2030 [4]. Few young doctors are willing to devote themselves to primary health care, and many of those doctors already in practice are leaving [2]. Therefore, greater emphasis should be placed on the retention of primary care doctors.

According to the theory of planned behaviour, behavioural intention is a good predictor of actual behaviour [5]. At present, turnover intention is believed to be a more revealing figure than the turnover rate because intention is the antecedent of resignation, and it has a better predictive ability [6, 7]. Turnover intention is used to measure people’s intention to resign from their current job and look for another job, and follow through on resigning [8]. Hom and Griffeth describe turnover intention as the relative strength of an individual’s desire to voluntary withdrawal from an organization [9]. Intention is the reason, and turnover is the result. The intention to resign leads to job searching behaviour, and when an alternative job presents itself, turnover occurs [10]. Steel and Ovalle reported a correlation coefficient of 0.50 between turnover intention and actual turnover after reviewing a significant number of studies [11]. A longitudinal study reported that family physicians in England with a strong intention to resign had 4.48 times the probability (OR = 4.48, 95%CI: 2.32–8.66, *p* < 0.001) of actual resignation after 5 years compared with physicians with no turnover intention [12]. These results indicate that higher turnover intention indeed predicted a greater likelihood of actually leaving. Accordingly, placing a focus on factors that affect the intention to resign rather than addressing the turnover itself would be more efficient [13, 14].

There are no formally validated scales that measure turnover intention [15]. Most researchers have adopted single-item scales to measure turnover intention [16, 17]. However, this kind of measurement had obvious metric limitations [18]. Michaels et al. developed a turnover intention scale [10], and Li et al. revised the scale for use in China, where it was used to measure nurses’ turnover intention. This revised scale, consisting of six items, included three aspects of turnover intention and surmised that an individual’s turnover intention depended on his or her intention to resign, motivation to search for a new job and the probability of finding another job [19, 20].

Factors associated with turnover intention, such as job satisfaction, working conditions and individual characteristics, have been reported in the literature [21]. Job satisfaction encompasses an employee’s level of satisfaction with the job and work environment [22]. Some researchers noted that job satisfaction was the most important antecedent variable for directly anticipating turnover intention [23, 24]. However, it is important to know which particular aspects of job satisfaction affects turnover intention. One study explored how satisfaction with the job rewards affected employees’ turnover intention. The researchers found that turnover intention was affected by satisfaction with financial rewards, material rewards and psychological rewards. Furthermore, along with job satisfaction, individual characteristics, such as gender, age and family responsibilities, also play an essential role in turnover intention [25].

In China, two types of doctors deliver primary care services: village-level doctors and township-level doctors. Village-level doctors are not considered government employees and only receive limited compensation from the government, and do not receive insurance coverage, while township-level doctors receive fixed wages and employee insurance [26]. In addition, working conditions for village-level doctors are poorer than those of township-level doctors. In China, the proportion of the PHC health workers in the health workforce decreased by 5.9% between 2009 and 2014 [27]. The number of PHC doctors falls short of the 1.25 million primary care doctors needed to ensure that China meets the goal established by the National Medical and Health Service System Planning (2015–2020), which requires that “number of primary care doctors per thousand population is 3.5 and above by 2020”.

In 2009, the Chinese government launched the Health System Reform Plan to strengthen PHC to improve the delivery of basic medical care and public health services [28]. However, the investment in the delivery side of PHC is insufficient, which may affect the retention of workforce [29]. A study from central China showed that income satisfaction and organizational policies affected the turnover intention of village-level doctors [30]. Another study in the capital of China noted that improving the retirement pensions and incomes of rural doctors helped increase human resources for PHC [26].

There are scarce data regarding turnover intention among PHC doctors in western China, which is the poorest regions and which has far less health resources than the rest of China. In this study, we conducted a sampling study of primary care doctors in Chongqing (southwestern China) with the aim of evaluating their level of turnover intention and exploring its associated factors. We hypothesized that (1) turnover intention was associated with job satisfaction and affected by some aspects of job satisfaction and (2) socio-demographic characteristics played a role in turnover intention.

## Methods

### Sampling and data collection

This study was conducted in Chongqing, which is located in southwestern China. Chongqing is one of the four municipalities under direct control of the central government of China, which occupies 82.4 thousand square kilometres with a population of 30.07 million (according to 2015 figures). In 2013, the government divided Chongqing into five areas based on their available socio-economic resources: the metropolitan core area, the metropolitan extension area, the newly developed urban area, the ecological conservation area and the environmental protection area.

Chongqing has become well developed in recent years and has received considerable attention from the central government of China; however, the primary health care situation is troubling. In 2014, the number of doctors per 1000 residents was only 1.72, the 10th lowest among the 12 provinces of western China. According to the Health Statistical Yearbook for Chongqing, the number of primary care doctors in township health centres decreased by 10.92%, and the number of health care workers in village clinics decreased by 10.58% between 2011 and 2016 [31]. The disparity in human resources among the different areas is also evident. In 2014, the metropolitan area had the highest doctor-patient ratio (2.78 doctors per thousand population), while the environmental protection area had the lowest (1.45 doctors per thousand population). To some extent, we may view Chongqing as being representative of China as a whole, since it includes both urban areas and remote poor villages. For example, the per capita gross domestic product (GDP) of Yuzhong district, which is located in the metropolitan core area, was more than 30 000 dollars, while the per capita GDP in Wuxi (a county in the ecological conservation area) was only 3014 dollars in 2015 [31]. A similar pattern of disparities in health care workers and economic development can be seen throughout the country. This study provides a chance to understand the problems that exist in the primary care system from the perspective of those that deliver healthcare services.

In this study, we combined the metropolitan core area and the metropolitan extension area into one metropolitan area, which allowed us to obtain a sample from each of the four areas with a complete administrative regional boundary descripted earlier. A multi-stage stratified random sampling method was used to collect a sample of 350 township-level doctors from 32 township health centres or community health centres, and 90 village-level doctors from 64 village clinics or community health stations. All the participating doctors gave their informed consent before completing the questionnaire.

### Measurement instruments

A self-administered questionnaire consisting of four parts was used for this quantitative survey; it included a cover letter outlining the objective of the study and the instructions on how to complete the survey.

- Part 1 included basic socio-demographic characteristics: gender, age, education background, and medical practice type, job title and location of job within the four previously described areas.
- Part 2 composed of work-related characteristics including the organization, amount of pressure at work, years of work, work hours per week, number of patients seen per week, annual income and training.
- In part 3, job satisfaction was measured using a 16-item questionnaire produced by Warr et al., we included an additional item to measure job satisfaction associated with doctor-patient relations (“relationship with patients”) [32]. Thus, we developed a 17-item questionnaire using a 7-point scale with responses ranging from 1 = “very dissatisfied” to 7 = “very satisfied”. Higher scores indicated a higher level of satisfaction on each item. The Cronbach’s alpha was excellent (0.96).
- Part 4 comprised multiple choice questions about reasons for resigning, as well as the six-item turnover intention scale (TIS-6), developed by Michaels et al. and revised by Li et al. for the Chinese population, which was used to measure the doctors’ intention to resign [10, 20]. Items included “Have you considered resigning from your current job?”, “Do you want to find another, similar job?” and four other items. A 4-point scale was adopted for each item with responses ranging from 1 = “never” to 4 = “always”. A high score indicated stronger turnover intention, and the total score of all six items was calculated to assess the overall turnover intention (“≤ 15” comprised the “low” group and “>15” comprised the “high” group). Items 1 and 6 were related to turnover intention I, indicating the intent to resign from the current job (TIS-I); items 2 and 3 were related to turnover intention II, indicating the motivation to search for other jobs (TIS-II); and items 4 and 5 were related to turnover intention III, indicating the probability of obtaining a new job (TIS-III). The Cronbach’s alpha of the TIS-6 was 0.75.

### Data analysis

Through double entry and validation in EpiData3.1, collected data were analysed using SAS 9.4 (SAS Institute, Inc., NC). Means and standard deviations (SD) were calculated for quantitative data, while percentages were calculated for qualitative data. Descriptive statistical analyses were conducted for socio-demographic variables, work-related characteristics, job satisfaction and turnover intention scores. *χ*^2^ test was used to compare the different reasons given for resigning from the current job by doctor’s position level (i.e. township or village level), age and location areas. One-way analysis of variance (ANOVA) was used to explore the impact of socio-demographic variables and work-related characteristics on doctors’ turnover intention. Exploratory factor analyses (EFA) was conducted for the job satisfaction scale using the principal component extraction method with an orthomax rotation to determine whether unique patterns of items existed. The principles including “the eigenvalue for each factor ≥1”, “cumulative variance explained by factors is above 75%” and scree plots were all taken into consideration when determining the common factors. The explanation of practical significance for each underlying factor was based on the factor loading value. A value of 0.5 is considered acceptable. Regression estimation was conducted to evaluate the factor score for each observation [33, 34]. In model I, significant socio-demographic variables in one-way ANOVA and overall job satisfaction were included, and in model II, the factors extracted by exploratory factor analysis replaced overall job satisfaction. The level of *p* < 0.05 was considered statistically significant in all tests.

## Results

### Socio-demographic and work-related characteristics of the study sample

A total of 440 primary care doctors participated in this survey, and 433 validated questionnaires were collected. There were 345 township-level doctors and 88 village-level doctors. Of these participants, 68.4% were male. The mean age was 40.9 (±9.93) age ranged from 21 to 72, 22.6% of the doctors had bachelor’s degrees or higher, and 27.0% had a mid-level or higher job title. The mean number of hours worked per week was 54.7 h (median = 50 h), and the mean number of patients seen per week was 62.9 (median = 50). Among the sample, 63.9% had an annual income of 20,000–39,999 RMB (2929–5858 US dollars), and 73.9% of the primary care doctors had participated in vocational training during the last 3 years (Table 1).

**Table 1:** Descriptive data for the primary care doctors who participated in this study (*n* = 433)

Socio-demographic and work-related factors	Groups	Number of doctors	Turnover intention	*p* value
		*n* (%)	($\overline{\boldsymbol{x}}\pm \boldsymbol{\delta}$)	
Doctor’s position level	Township level	345(79.7)	14.64 **±** 3.43	*< 0.0001*
	Village level	88(20.3)	12.61 **±** 4.17	
Location areas	Metropolitan area	101(23.3)	12.54 **±** 3.31	*< 0.0001*
	Newly developed urban area	106(24.5)	14.67 **±** 3.46	
	Ecological conservation area	121(27.9)	14.14 **±** 3.72	
	Environmental protection area	105(24.3)	15.50 **±** 3.62	
Medical practice type	Traditional Chinese medicine	59(13.4)	13.42 **±** 3.82	*0.0498*
	Western medicine	196(45.3)	14.65 **±** 3.56	
	Integrated traditional Chinese and Western medicine	178(41.1)	14.02 **±** 3.72	
Gender	Male	296(68.4)	14.34 **±** 3.84	0.3548
	Female	137(31.6)	13.99 **±** 3.32	
Age	< 30	54(12.5)	14.17 **±** 3.32	*0.0025*
	30-39	145(33.5)	15.06 **±** 3.49	
	>=40	234(54.0)	13.72 **±** 3.80	
Education	Bachelor’s degree or higher	98(22.6)	14.69 **±** 3.36	*0.0009*
	College degree	202(46.7)	14.65 **±** 3.62	
	High school graduation (secondary technical school) or below	133(30.7)	13.24 **±** 3.84	
Job title	None	61(14.1)	13.13 **±** 4.06	*0.0177*
	Entry level	255(58.9)	14.23 **±** 3.84	
	Middle level or above	117(27.0)	14.78 **±** 2.96	
Years of working	< 10	84(19.4)	14.24 **±** 3.43	*0.0139*
	10-19	159(36.8)	14.84 **±** 3.47	
	>=20	189(43.8)	13.68 **±** 3.89	
Annual income	< 20 000	66(15.3)	14.26 **±** 4.12	0.9854
	20 000-39 999	276(63.9)	14.22 **±** 3.66	
	>=40 000	90(20.8)	14.30 **±** 3.33	
Working time per week	< 50	164(37.9)	13.37 **±** 3.47	*0.0001*
	>= 50	269(62.1)	14.75 **±** 3.71	
Number of patients per week	< 50	214(49.4)	14.07 **±** 3.85	0.3692
	>=50	219(50.6)	14.38 **±** 3.51	
Training during the last 3 years	Yes	320(73.9)	14.08 **±** 3.75	0.1502
	No	113(26.1)	14.65 **±** 3.45	

Note: figures in italics mean *p* < 0.05, indicating that the results of one-way ANOVA showed statistical significance

### Turnover intention and related reasons among primary care doctors

**Fig. 1:**
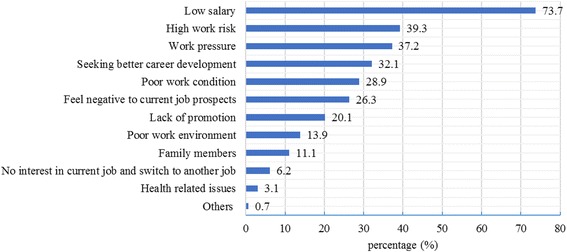
Main reasons to quit current job

**Fig. 2:**
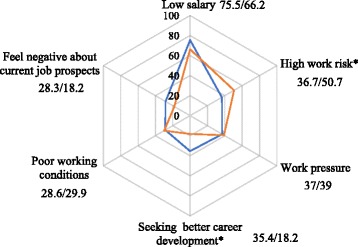
Reason to quit current job by doctor’s position level. Blue: township-level doctor (%). Orange: village-level doctor (%). Note: (1) * means *p* < 0.05, indicating that the results of *χ*^2^ test showed statistical significance; (2) the number near to each reason represent percentage for township-level doctor and village-level doctor respectively

**Fig. 3:**
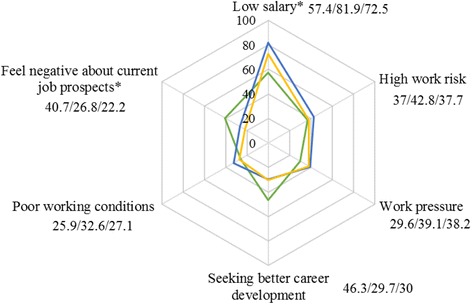
Reason to quit current job by age. Green: Age < 30 (%). Blue: Age 30-39 (%). Yellow: Age >=40 (%). Note: (1) * means *p* < 0.05, indicating that the results of χ^2^ test showed statistical significance; (2) the number near to each reason represent percentage for primary care doctors age under 30 years old, age between 30 and 39 years old and age 40 years old or older respectively

**Fig. 4:**
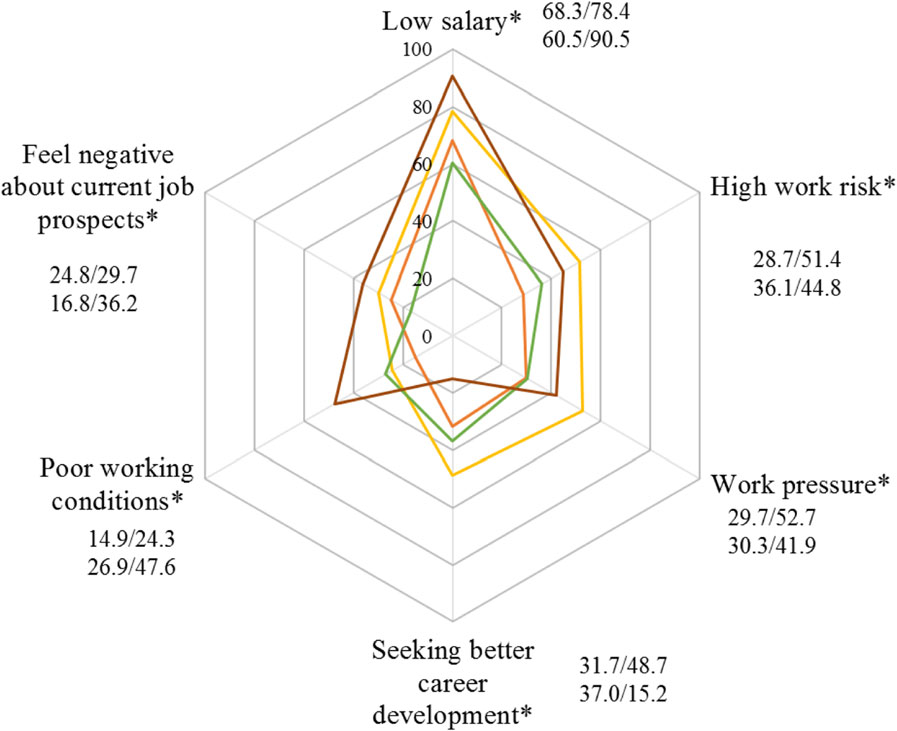
Reason to quit current job by location areas. Orange: metropolitan area (%). Yellow: newly developed urban area (%). Green: ecological conservation area (%). Brown: environment protection area (%). Note: (1) * means *p* < 0.05, indicating that the results of *χ*^2^ test showed statistical significance; (2) the number near to each reason represent percentage for metropolitan area, newly developed urban area, ecological conservation area and environment protection area respectively

The mean total score of turnover intention among the participants in this survey was 14.23 ranged from 6 to 24. The probability of obtaining a new job (TIS-III) had the highest score (mean score = 5.67 ranged from 2 to 8), followed by motivation to search for other jobs (TIS-II; mean score = 4.31 ranged from 2 to 8); intention to resign from the current job (TIS-I) had the lowest score (mean score = 4.25 ranged from 2 to 8). Based on the criterion of scores “>15” as “high”, 42.3% of the doctors had high turnover intention. The main reasons for resigning from the current job included “low salary” (73.7%), “high work risk” (39.3%), “work pressure” (37.2%) and “seeking better career development” (32.1%) (Fig. 1). About half (50.7%) of the village-level doctors regarded high work risk as the main reason to resign, which was higher than township-level doctors (36.7%), while more township-level doctors (35.4%) than village-level doctors (18.2%) gave “seeking better career development” as the main reason to resign (Fig. 2). Eighty one point nine percent of primary care doctors aged from 30 to 39 years thought that salary was the main reason to resign, and more doctors under the age of 30 (40.7%) thought that current job prospects was the main reason to resign (Fig. 3). Figure 4 shows that more primary care doctors working in the environmental protection area considered low salary (90.5%), poor working conditions (47.6%) and current job prospects (36.2%) as the main reasons to resign, while more primary care doctors in the newly developed urban area considered work pressure (52.7%), high work risk (51.4%) and seeking better career development (48.7%) as the main reasons to resign. All the differences mentioned above were statistically significant (*p* < 0.05).

### Job satisfaction among primary care doctors and exploratory factor analysis of the job satisfaction scale

The mean score of overall job satisfaction of the primary care doctors was 4.89 ranged from 1 to 7. The doctors were most dissatisfied with “your rate of pay” (score = 3.61 ± 1.66 ranged from 1 to 7), followed by the chance of job promotion (score = 4.22 ± 1.54 ranged from 1 to 7). The top four common factors on satisfaction were extracted from the job satisfaction scale with a cumulative variance of 78.7%. The communality variance for each item was greater than 70%, which means that these four factors reflect the most information for each item. According to the 0.5 principle, factor 1 included “your immediate boss” and four other items, reflecting *satisfaction with organizational administration*. Factor 2 included “your chance of promotion”, “your rate of pay”, and two other items, reflecting *employment-related job satisfaction*. Factor 3 included “the freedom to choose your own method of working”, “your job safety” and two other items, reflecting *satisfaction with the job itself*. Factor 4 included “your fellow workers” and two other items, indicating *relationship-related satisfaction*. The factor loading distribution map is shown in Table 2.

**Table 2:** Score and exploratory factor analysis results for the job satisfaction scale (*n* = 433)

Items	Score ($\overline{\boldsymbol{x}}\pm \boldsymbol{\delta}$)	Factor 1	Factor 2	Factor 3	Factor 4	Communalities
Your immediate boss	5.38 **±** 1.38	0.819				0.839
Industrial relationship between management and workers in your firm	5.03 **±** 1.31	0.704				0.828
The attention paid to suggestions you make	4.60 **±** 1.30	0.659				0.805
The way your firm is managed	4.69 **±** 1.40	0.637				0.748
The amount of responsibility you are given	4.86 **±** 1.33	0.635				0.746
Your chance of promotion	4.22 **±** 1.54		0.830			0.821
Your rate of pay	3.61 **±** 1.66		0.790			0.774
Your opportunity to use your abilities	4.56 **±** 1.35		0.624			0.749
Your hours of work	4.46 **±** 1.44		0.555			0.725
The freedom to choose your own method of working	4.38 **±** 1.46			0.724		0.798
Your job safety	4.88 **±** 1.32			0.643		0.788
The amount of variety in your job	4.45 **±** 1.33			0.634		0.805
The physical working conditions	4.40 **±** 1.44			0.571		0.798
Your fellow workers	5.12 **±** 1.25				0.716	0.822
The recognition you get for good work	4.82 **±** 1.25				0.681	0.813
Relationship with patients	5.09 **±** 1.19				0.550	0.732
Now, taking everything into consideration, how do you feel about your job as a whole?	4.89 **±** 1.29					
Eigenvalue		9.998	1.162	0.779	0.652	
Cumulative variance explained by factors (%)		0.625	0.698	0.746	0.787	

### Predictors of turnover intention among primary care doctors

One-way ANOVA assesses the association between the turnover intention and socio-demographic and working-related variables (Table 1), and these variables with statistical significance are included in the regression analysis.

Controlling for other variables, doctor’s position level was an important predictor of turnover intention (*b* = − 0.174 in model I; *b* = − 0.138 in model II). Township-level doctors had higher turnover intention than village-level doctors. Similarly, location areas were another predictor of turnover intention. Compared with the metropolitan area, doctors in the other three areas showed higher turnover intention, especially in the newly developed urban area (*b* = 0.264, *p* < 0.0001) and the environmental protection area (*b* = 0.263, *p* < 0.0001). Younger doctors had higher turnover intention; doctors with higher job titles had higher turnover intention; and higher work pressure predicted higher turnover intention. In model I, overall job satisfaction was negatively associated with turnover intention (*b* = − 0.180, *p* < 0.0001). When four job satisfaction factors replaced overall job satisfaction, the adjusted *R*^2^ changed from 0.257 to 0.303. Factor 2 (*employment-related job satisfaction*) and factor 3 (*satisfaction with the job itself*) significantly predicted turnover intention among primary care doctors. Items such as “your chance of promotion (factor loading = 0.830)”, “your rate of pay (factor loading = 0.790)”, “the freedom to choose your own method of working (factor loading = 0.724)” and “your job safety (factor loading = 0.643)” had high factor loading (Tables 2 and 3).

**Table 3:** Multivariate linear stepwise regression analysis of factors related to turnover intention

Factors	Model I		Model II	
	*b*	*p*	*b*	*p*
Age, years				
< 30 vs >= 40	0.074	0.1151	0.107	0.0228
30-39 vs >=40	0.179	0.0001	0.208	< 0.0001
Location areas				
Newly developed urban area vs metropolitan area	0.264	< 0.0001	0.248	< 0.0001
Ecological conservation area vs metropolitan area	0.146	0.0078	0.116	0.0342
Environment protection area vs metropolitan area	0.263	< 0.0001	0.222	< 0.0001
Doctor’s position level				
Village-level doctors vs township-level doctors	− 0.174	0.0001	− 0.138	0.0031
Work pressure	0.241	< 0.0001	0.196	< 0.0001
Job title	–	–	0.109	0.0176
Overall job satisfaction	− 0.180	< 0.0001	–	–
Factor 2	–	–	− 0.297	< 0.0001
Factor 3	–	–	− 0.142	0.0015
*R*^2^	0.271		0.320	
Adjusted *R*^2^	0.257		0.303	

## Discussion

Strengthening PHC has high priority in China’s new medical system reform. Reducing primary care doctors’ turnover intention and improving the stability of human resources for PHC is crucial to the success of the reforms. We found that 42.3% of primary care doctors in the study had high turnover intention, which is higher than the rate of community health practitioners in the five other provinces in China (38.7%) [35] and higher than village doctors in Xiangyang, a city in Hubei province of China (36.8%) [30], while a study from England showed that only 11.8% of primary care doctors had high turnover intention [12]. In addition, the score of turnover intention III (the probability of obtaining a new job) was the highest compared with the two other dimensions, probably due to the increase in available jobs as a result of the new medical reform. While the new medical reform aims to improve PHC, it unfortunately creates a brain drain as doctors move away from primary care practice [36]. The reform of public hospitals at the county level has expanded the scale of medical institutions, and a policy that encourages private investment in the medical field has improved the medical market share of private hospitals. Senior medical facilities, including county public hospitals and private hospitals, are willing to recruit experienced doctors from primary care settings [36]. In this survey, we also found socio-demographic variables such as working at the township level, having a higher job title or being between 30 and 39 years of age, were associated with higher turnover intention, probably due to their greater experience and professional ability, and higher probability of changing a job.

In this study, we found a negative correlation between job satisfaction and turnover intention among primary care doctors, which is in line with similar reports [16, 17, 30, 35]. Using exploratory factor analysis, we integrated the 17 items of the job satisfaction scale into four core factors, of which *employment-related job satisfaction* and *satisfaction with the job itself*, including chance of promotion, your rate of pay and the other six items, were the two that significantly predicted turnover intention. Those items were correlated and interactional affecting turnover intention.

Of the eight items of job satisfaction, chance of promotion and your rate of pay had the highest factor loading, as knowledgeable doctors, especially young doctors, pay more attention to career development when choosing a job, including the chance of promotion and training opportunities [37]. Compared with large public hospitals, the working conditions of primary care facilities are poorer, with fewer training opportunities and limited chances for job promotion [36, 38]. In this study, doctors under 30 years old were more likely to think that seeking better career development opportunities (46.3% of doctors) and feeling negative about prospects in their current positions (40.7% of doctors) were the main reasons for resigning from a job. Both these rates were lower than 30% in doctors older than 30 years of age. Meng et al. also reported that primary care doctors transferred to higher-level medical institutions because of limited opportunities for job promotion in primary care facilities [39]. Less than 25% of the doctors who participated in this study had a bachelor’s degree or higher, and over 70% of them had only an entry level job title or lower. These results reflect both the shortage of high-level doctors in PHC and the difficulty of professional advancement, both of which are due to inadequate training mechanisms and job title evaluation mechanisms. Although primary care doctors are willing to participate in training or on-the-job education to improve their professional abilities, training is expensive and income during training is not always guaranteed. Therefore, the training is mostly short-term or a mere formality, and this may be the reason no association between training and turnover intention was found in our study, which was different from the findings of other studies [35, 40–42]. Due to the lack of effective training, it is difficult for primary care doctors to improve their professional abilities. In short, few opportunities and strict evaluations lead to limited job promotion for doctors in primary care facilities [36].

Low salary, which is disproportionate to the high workload and work pressure, is one of the main reasons for high turnover intention. After the Chinese government launched the Health System Reform Plan in 2009, the content of basic public health services was expanded, and primary care doctors are now required to deliver both medical services and public health services [28]. If the PHC workforce is not significantly increased, this demand can only be met by increasing the workload of primary care doctors, which will undoubtedly increase both the physical and psychological pressure they experience [29]. We found that 62.1% of the doctors in this survey worked more than 50 h per week. The reform of human resources for primary care facilities is still underway, and the mechanism for incentive and compensation are still imperfect. PHC doctors’ income has been affected by the Chinese government’s a zero-mark-up policy on drug sales. For one thing, the profits from drug sales have disappeared; for another thing, patients may go to other medical institutions because of the limitation of the varieties of basic drugs available at primary care facilities; however, the financial support is not subsidized timely. In one study, 74.8% of village-level doctors reported dissatisfaction with their financial compensation [30]. Another study reported that primary care doctors thought that their salaries were disproportionate to their amount of work [43]. This kind of inequality causes job dissatisfaction, which affects turnover intention [33]. Among primary care doctors between 30 and 39 years old, and who are the primary breadwinners in their households, 81.9% thought the main reason to resign was low salary, compared with only 57.4% of those under the age of 30.

Job safety also has a high factor loading. Low job safety predicts high turnover intention. At present, the medical environment is deteriorating, and medical disputes are continuing to increase, posing threats to doctors’ personal safety [44]. Due to the low education level and limited professional ability of primary care doctors, the feeling of job unsafe is even more serious, especially among village-level doctors. One explanation is that most village-level doctors are private practitioners, whose job safety is not safeguarded by large institutional and rules and regulations [45]. In addition, they do not have fixed wages and the same social welfare benefits as township-level doctors or even village teachers, causing village-level doctors to feel unsafe and unsatisfied with their job. [46]. We found that 50.7% of the village-level doctors thought that high risk to their safety at work was the main reason to resign from a job, compared with 36.7% of the township-level doctors.

In addition to the chance of promotion, your rate of pay and job safety, as mentioned above, the freedom to choose their own method of working, physical working conditions and the amount of variety in their job also affected turnover intention. Wu et al. reported the deteriorating doctor-patient relationships had an impact on doctors’ job satisfaction [44]; however, in our study, the primary care doctors were satisfied with their doctor-patient relationships, and there was no association found between doctor-patient relationship and turnover intention. In Chinese town and village culture, the relationship between primary care doctors and patients is closer than a typical doctor-patient one which promotes deep mutual trust and communication due to their shared family, communities, lifestyles and beliefs [47].

Location areas also matter significantly in explaining turnover intention. Doctors in the environmental protection area had the highest turnover intention. This area is least-developed with inferior primary care facilities, and most of the doctors working there reported that low salary (90.5%) and poor working conditions (47.6%) were the main reasons for turnover intention, these rates were much higher than that of other areas. Doctors in the newly developed urban area also had high turnover intention, possibly because of the rapid urbanization process in this area. On the one hand, fast urbanization results in a series of social problems (e.g. larger population, heavy traffic, increasing air pollution), which may have an impact on people’s health. For another thing, the growth rate of investment in human resources for health lags considerably behind population growth and urbanization progress [48], which can result in additional workload and pressure for primary care doctors, thus leading to high turnover intention. In the newly developed urban area, primary care doctors thought that work pressure (52.7%) and high work risk (51.4%) were the main reasons for turnover intention, which was higher than the other three area locations. On the other hand, urbanization also provides primary care doctors with more opportunities to seek better career development, and 48.7% of the doctors in the newly developed urban area reported seeking better career development as the main reason for turnover intention.

The Chinese government has taken action to address the shortage of health care workers for PHC, especially in rural areas. For example, in 2010, the Chinese government launched a “rural-oriented medical student cultivation programme” [49]. However, the effect of this policy intervention was not ideal. Several studies have shown that 35.28% of enrolled students wanted to default after graduation, and less than 5% of students were willing to continue to deliver PHC services after the expiration of their agreed mandatory time [50–52]. Therefore, the key to reducing the turnover intention of primary doctors and solving the shortage of human resources for PHC is to improve the working and living conditions of primary care doctors and increase their chance of promotion.

### Limitations

The first limitation of this study is the representativeness of the sample. Our research sample was relatively small, which may have an impact on statistical reference and test power. However, we collected the sample using a strict sampling method, and the participants were well-distributed throughout Chongqing, which can guarantee the sample’s representativeness. Second, there may be information bias resulting from social proof or “correctness” since we used a questionnaire in this quantitative research; however, the bias has been greatly reduced by making the participants anonymous, a well-designed questionnaire and good communication between investigators and participating doctors. Third, this cross-sectional study limited the range of information we could gather. Therefore, in future research, we will expand the sample size and conduct a longitudinal study to evaluate turnover intention and the relationship between intent to resign and turnover.

## Conclusions

Primary care doctors play a vital role in driving the performance of health care systems at the grassroots level. Our study showed that the turnover intention of primary care doctors in Chongqing, China, is high and is affected by different aspects of job satisfaction. Considering that improving job satisfaction, in terms of salary, promotion and job safety, is crucial for reducing turnover intention among primary care doctors, we suggest that the government increase its financial investment in primary care facilities, especially in less-developed areas, and reform incentive mechanisms to improve the job satisfaction of primary care doctors. The government should consider policies such as establishing a social pension programme and legal protection system for village-level doctors to reduce their vulnerability to medical risks, improving training mechanisms and establishing a job title evaluation system to increase the chance of job promotion among primary care doctors, especially young doctors and township-level doctors. Attention should also be paid to the impact of rapid urbanization, which could lead to increased workload or increased opportunities for career development, thus affecting primary care doctors’ turnover intention. The adoption of these recommendations may help to accelerate the development of PHC in Chongqing, China.

## Abbreviations

ANOVA: Analysis of variance
*b*: Standardized regression coefficient
EFA: Exploratory factor analysis
GDP: Gross domestic product
PHC: Primary health care
*p* value: Level of significance
SD: Standard deviation
TIS-6: Turnover intention scale
TIS-I: Turnover intention I
TIS-II: Turnover intention II
TIS-III: Turnover intention III
*χ*^2^: Chi-square test
